# Small airways in asthma: Pathophysiology, identification and management

**DOI:** 10.1016/j.pccm.2023.07.002

**Published:** 2023-09-13

**Authors:** Dimitrios Toumpanakis, Omar S. Usmani

**Affiliations:** 1 National Heart and Lung Institute, Imperial College London, London, SW3 6LY, United Kingdom; 2 General State Hospital for Thoracic Diseases of Athens “Sotiria”, Athens, 11527, Greece

**Keywords:** Small airways, Asthma, Oscillometry, Nitrogen washout

## Abstract

**Background:**

The aim of this review is to summarize the current evidence regarding small airway disease in asthma, focusing on recent advances in small airway pathophysiology, assessment and therapeutic implications.

**Methods:**

A search in Medline was performed, using the keywords “small airways”, “asthma”, “oscillometry”, “nitrogen washout” and “imaging”. Our review was based on studies from adult asthmatic patients, although evidence from pediatric populations is also discussed.

**Results:**

In asthma, inflammation in small airways, increased mucus production and airway wall remodelling are the main pathogenetic mechanisms of small airway disease. Small airway dysfunction is a key component of asthma pathophysiology, leading to increased small airway resistance and airway closure, with subsequent ventilation inhomogeneities, hyperresponsiveness and airflow limitation. Classic tests of lung function, such as spirometry and body plethysmography are insensitive to detect small airway disease, providing only indirect measurements. As discussed in our review, both functional and imaging techniques that are more specific for small airways, such as oscillometry and the multiple breath nitrogen washout have delineated the role of small airways in asthma. Small airways disease is prevalent across all asthma disease stages and especially in severe disease, correlating with important clinical outcomes, such as asthma control and exacerbation frequency. Moreover, markers of small airways dysfunction have been used to guide asthma treatment and monitor response to therapy.

**Conclusions:**

Assessment of small airway disease provides unique information for asthma diagnosis and monitoring, with potential therapeutic implications.

## Introduction

**Fig. 1:**
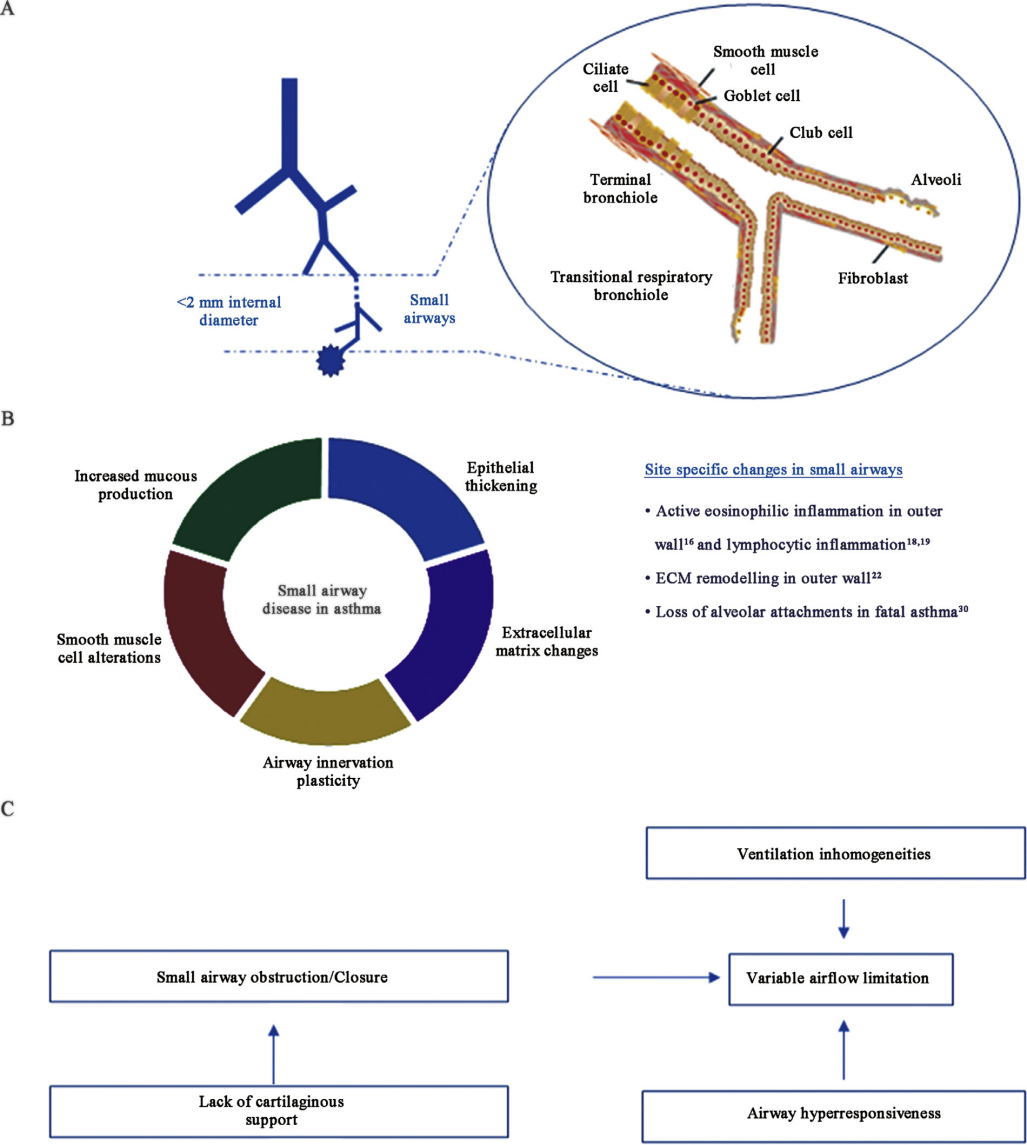
Overview of small airway disease pathogenesis and pathophysiology in asthma. (A) Small airways, i.e., airways with internal diameter of <2 mm, consist of small conducting (terminal) bronchioles, respiratory bronchioles and alveolar ducts, and are characterized by the lack of cartilage in their wall (modified from Ref. 128 with permission). (B) In asthma, mainly due to airway inflammation, increased mucous production, epithelial thickening, extracellular matrix remodelling, neuronal plasticity, and smooth muscle cell hyperplasia/hypertrophy lead to the development of small airway disease. Spatial differences between larger and small airways exist, since for example increased numbers of activated eosinophil are found in the small airways, located in the outer wall of the airways. (C) The net effect of the pathogenetic mechanisms of small airway disease, assisted by the lack of cartilaginous support, leads to the development of the classic triad of asthma pathophysiology, i.e., airway obstruction/closure, ventilation inhomogeneities and hyperresponsiveness, resulting in airflow limitation.

Asthma is a heterogenous disease,^1^ characterized by reversible airflow limitation and symptoms of cough, dyspnea, wheezing and chest tightness.^2^ Small airways, i.e., the peripheral airways with diameter less than 2 mm (Fig. 1A), are the main site of increased airway resistance in asthma^3^ and numerous studies have correlated small airway disease with clinical outcomes, including asthma severity, frequency of exacerbations and response to therapy.^4^^,^^5^ Despite the cumulative evidence for the role of small airway dysfunction in asthma, the assessment of small airways is undervalued in current daily clinical practice.^6^ For example, the European Respiratory Society guidelines on the diagnosis of asthma have not included small airway assessment in the official diagnostic work-up,^7^ and no statement regarding small airway disease is made in the Global Initiative for Asthma (GINA) report and severe asthma guide.^2^ The above may reflect the lack of standardization of small airway function techniques and the scarcity of large clinical trials that include small airway disease markers as trial endpoints.

Of note, recent publications of studies regarding small airway disease in asthma and the European Respiratory Society initiatives on standardization of techniques focusing on small airway function,^8^, ^9^, ^10^ may foster the implementation of small airway function in future asthma diagnosis and treatment guidelines. Thus, the aim of our review is to summarize current knowledge on small airway disease in asthma, focusing on recent advances in small airway pathophysiology, assessment, and role in therapeutic management.

## Methodology

We searched Medline for the terms of “small airways”, “asthma”, “oscillometry”, “nitrogen washout”, “imaging”, including both original research papers and reviews. Mainly studies with adult asthmatic patients were included in our review, although some evidence from pediatric populations is also discussed. No time restrictions were applied, although our review is focused on recent advances in the field. The initial set of relevant papers was shortlisted to those of interest, based on the opinion and expertise of the authors. Only papers written in English were used. It is worth mentioning that the topic of “small airways in asthma” has gained great attention over the last decades, given that a search with this phrase retrieved 704 results between 1980 and 1999, while 2603 articles were retrieved between 2000 and 2022. Thus, we acknowledge that not all relevant studies could be included in a single review, despite the effort made not to omit any significant findings on the field.

## Pathogenesis of small airway disease in asthma

Small airway disease in patients with asthma is the net effect of alterations in various compartments of the small airway wall, including increased mucus production and goblet cell metaplasia, epithelial shedding, variable thickening of pulmonary epithelium with reticular basement membrane thickening, smooth muscle cell layer hyperplasia and hypertrophy, and changes in extracellular matrix (ECM) composition (Fig. 1B).^11^ Although several factors mediate changes in airways in asthma, such as genetic predisposition^12^ and oxidative stress,^13^ chronic airway inflammation is the central process mediating small airway disease and remodelling.^14^ Moreover, inflammation leads to plasticity in both afferent and efferent limb of the airway innervation, inducing increased cholinergic tone and contributing to asthmatic phenotype.^15^

Interestingly, the inflammatory process in the airway tree is shown to differ between large versus small airways and across different degrees of asthma severity. In 6 patients with asthma, histological analysis of lung tissue samples revealed that while both central and peripheral (<2mm diameter) airways were infiltrated by T cells and eosinophils, active eosinophilic inflammation was higher in small airways and was localized in the outer airway layer (between smooth muscle layer and adjacent parenchyma).^16^ In the same cohort of patients, increased expression of interleukin (IL)-5 was detected in small airways, in contrast to more proximal central airways.^17^ Similarly, Kraft et al^18^^,^^19^ reported that in patients with nocturnal worsening of asthma, infiltration of lung periphery with eosinophils and lymphocytes was noticed, which was correlated with worsened lung function, in contrast to eosinophilic inflammation in proximal airways. Haley et al^20^ have reported a differential distribution of inflammation in large versus small airways in patients with fatal asthma, with lymphocytes and eosinophils distribution in the outer wall of the small airways, thus in close proximity to alveolar attachments, where inflammation can be spread.

Spatial differentiation across the airway wall has also been observed for ECM remodelling. In both mild^21^ and severe asthma,^22^ significant ECM remodelling in small airways has been found, e.g., increased collagen deposition. Similarly, with respect to inflammatory changes in severe asthmatic patients, the outer wall of small airways has been shown to be the main site of ECM remodelling.^22^ Alterations in the airway smooth muscle cell layer is a major determinant of airflow limitation and hyperresponsiveness in patients with asthma. Although increased muscle mass is found in the airways of asthmatic patients and has been attributed to both hyperplasia and hypertrophy of muscle cells, the relative contribution of these phenomena, as well as the spatial characteristics (large *vs.* small airways) is still under active investigation with contradictory results in literature.^11^^,^^23^

## Pathophysiological consequences of small airway dysfunction in asthma

As mentioned above, asthma is a complex disease, comprised of various endotypes and clinical phenotypes.^1^ Irrespective, however, from the underlying mechanisms, the pathophysiology of asthma follows a common end pathway, characterized by increased airway resistance and airway closure, together with increased airway hyperresponsiveness and peripheral ventilation inhomogeneities, which leads to airflow limitation that is variable in nature, hyperinflation (gas trapping) and an increased work of breathing [Fig. 1C].^24^^,^^25^ Although describing the exact mechanisms that mediate each of these features is beyond the scope of this review, it is worth noticing that small airway disease contributes independently to each of the single components of asthma pathophysiology, indicating a significant role of small airways in asthma. Indeed, as stated previously, small airways are the main site of airway obstruction in asthma^3^ and in asthma patients, the presence of small airway disease was associated with increased prevalence of persistent airflow limitation.^26^

In small airways, the lack of cartilaginous support, together with the thickening of the airway wall, renders the airways more prone to obstruction and closure.^24^ Interestingly, pulmonary surfactant is present in the small airways epithelial lining fluid in addition to the alveoli, preventing airway closure. It has been shown that asthmatic airway inflammation disrupts optimal surfactant function,^27^ although nebulized surfactant failed to improve lung function in asthmatic children following histamine challenge.^28^ It is worth mentioning that the pathological alterations of small airways in asthma may have opposing effects in airway–parenchymal interdependence and airway constriction.^24^ On one hand, stiffening of airway wall from remodelling and subepithelial fibrosis may counteract smooth muscle contraction,^29^ on the other hand, airway wall edema and thickening promote airway constriction. Moreover, in fatal asthma, loss of alveolar attachment of small airways has also been observed,^30^ which may account for the exaggerated airway constriction, due to loss of airway parenchymal interdependence.^25^

Ventilation heterogeneity (VH) is a central component of asthma pathophysiology. Indeed, VH has been shown to be independently correlated with airway hyperresponsiveness, even when airway inflammation is included in multi-regression models.^12^ While the pathology in both large and small airways can cause VH,^25^ a recent study, using functional computed tomographic (CT) lung imaging, showed that small airway disease in lower lobes is the major determinant of ventilation heterogeneity in asthma patients.^31^

## Small airway assessment in asthma

Taking into consideration the important role of small airway disease for asthma pathogenesis and pathophysiology, techniques that evaluate small airway structure and function are in need. Lung histology that provides definite information on small airway disease, requires invasive procedures, such as transbronchial biopsies, which are not justified in daily asthma assessment, due to adverse effects. Indeed, even in severe asthma, obtaining lung tissue samples, outside of research purposes, remains controversial and not included in current guidelines.^7^^,^^32^^,^^33^ Unfortunately, classic tools of lung function, such as spirometry, are insensitive to detect small airway disease.^34^ Traditionally, the maximal mid expiratory flow (forced expiratory flow at 25–75% of forced vital capacity [FVC], FEF_25%-75%_) is considered a marker of small airway disease, nevertheless, its wide variability and influence by forced vital capacity (FVC), limit its clinical utility. Indeed, in a very large observational study, where the majority of patients were investigated for asthma diagnosis and/or control, adding FEF_25%-75%_ to the conventional spirometric indices (forced expiratory volume in one second [FEV_1_], FVC and FEV_1_/FVC ratio) offered additional clinical information in only a minority of patients.^35^ The difference between FVC and slow vital capacity (SVC) is shown to reflect airway collapse that could be attributed to small airways disease and/or emphysema^36^ and indeed, the ratio of FVC to SVC has been used as a marker of small airways dysfunction in patients with bronchiolitis obliterans syndrome.^37^ Gibbons et al^38^ showed that the percent fall in FVC during a bronchial challenge correlates with small airway disease in patients with mild asthma. Similar to spirometry, body plethysmography provides only indirect evidence for the presence of small airway disease in asthmatic patients. In asthmatic patients with nocturnal symptoms, residual volume (RV) measured by body plethysmography correlated with peripheral airway resistance, as measured by bronchoscopy.^39^ Moreover, the ratio of RV to total lung capacity (TLC), a marker of gas trapping, was increased in patients with severe asthma in contrast to mild asthma.^40^ Interestingly, almost half of asthmatic patients receiving inhaled corticosteroid (ICS)/long-acting beta-agonist (LABA) treatment without airflow obstruction in spirometry (normal FEV_1_/FVC and FEV_1_) showed evidence of small airway obstruction, as defined by increased FEF_25%–75%_ and SVC–FVC or by signs of lung hyperinflation in plethysmography (increased RV/TLC or functional residual capacity [FRC]), with the latter most commonly found.^41^

Fortunately, over the last decades, technological advances have enabled the commercial availability of novel lung function tools and the development of imaging techniques that allow a more comprehensive and specific assessment of small airways. Over the next paragraphs, techniques of small airway assessment will be described (summarized in Table 1), focusing on asthma, and the readers are also referred to extensive reviews in literature.^4^^,^^42^

**Table 1:** Techniques for the assessment of small airway disease and dysfunction in asthma.

Technique	Markers	Advantages	Disadvantages	Ref.
Lung histology	Histological features of small airway disease	Gold standard method for the detection of small airway pathology.	Requires invasive tissue sampling, not applicable to usual daily clinical practice. Restricted to research.	^33^
Spirometry	FEF_25%–75%_, SVC–FVC, FVC percent fall during bronchial challenge	Wide availability in various clinical settings; standardized criteria.	Non-specific markers for small airway dysfunction.	^35^^,^^36^^,^^38^
Body plethysmography	RV, RV/TLC	Detection of hyperinflation and gas trapping; widely available.	Indirect evidence of small airway involvement.	^40^
Oscillometry	Resistance (R_5_–R_20_), reactance (X_5_, A_x_), within-breath analysis	Requires minimal co-operation; performed during quietly breathing; more specific and sensitive to small airways; enables early diagnosis, assessment of bronchodilator response feasible.	Requires standardization and robust reference equations, absence in current diagnostic algorithms and treatment guidelines.	^9^^,^^46^^,^^48^^,^^49^^,^^51^
Inert gas washout (single- and multi-breath)	Slope of phase III (single breath), S_acin_, S_cond_, LCI (multi breath)	Detects ventilation heterogeneity and enables partition to proximal and peripheral lung abnormalities; sensitive to early disease.	Not widely available, used mainly for research purposes.	^62^^,^^64^^,^^66^
Computed tomography	Mosaic pattern, centrilobular nodules, functional parameters of small airway disease, expiratory to inspiratory lung density	Allows spatial distribution; useful in differential diagnosis; advanced algorithms also provide information on drug deposition; expected to play significant role in future.	Exposure to ionizing radiation; direct visualization of small airways not yet feasible; advanced imaging algorithms not widely available.	^31^^,^^69^^,^^70^
Hyperpolarized magnetic resonance imaging	Detection of ventilation distribution	No radiation exposure.	Limited availability; restricted to research.	^74^^,^^75^
Endobronchial optical coherence tomography	Direct visualization of small airways and/or alveoli.	Promising technique.	Requires bronchoscopy; not widely available; needs further validation.	^77^^,^^78^^,^^81^
Nuclear medicine techniques	Ventilation and/or radiolabeled molecules distribution in the lung.	Estimation of the spatial distribution of inhaled drugs and/or “target” receptors.	Exposure to radiation; difficult to isolate small airways; not widely available.	^84^
Biomarkers	F_E_NO	Phenotyping eosinophilic inflammation; partitioning to bronchial and peripheral NO production feasible; portable analyzers available; may aid therapeutic decisions.	Affected by smoking and ICS therapy.	^7^^,^^85^, ^86^, ^87^

FEF_25%–75%_: Maximal mid expiratory flow; FeNO: Nitric oxide (NO) in exhaled breath; FVC: Forced vital capacity; ICS: Inhaled corticosteroid; LCI: Lung Clearance Index; RV: Residual volume; S_acin_: Inhomogeneities created in the diffusion-convection front, i.e., the zone where flow is converted from convection to diffusion; S_cond_: Inhomogeneities created in the conducting airways, i.e., airways proximal to terminal bronchioles; SVC: Slow vital capacity; TLC: Total lung capacity.

## Functional assessment

### Oscillometry

Although respiratory physiologists have been investigating oscillatory mechanics of the lung since 1950s,^43^ oscillometry has recently gained much attention, particularly with the increased availability of modern devices, some of which are portable, and the increasing recognition that small airways are a major component of obstructive airway disease initiation and progression.^8^ Oscillometry consists of the application of a pressure signal of varying frequencies, superimposed on tidal breathing, while no co-operation is required, except for the fact that the subject supports their cheeks with their hands. Total respiratory impedance across the frequency spectrum is then mathematically derived and partitioned to resistance (R_rs_) and reactance (X_rs_), the latter consisting of the effect of elastance and inertance.^44^ Small airway disease alters airway resistance and reactance in a frequency-dependent manner, i.e., both R_rs_ and X_rs_ are more affected at lower frequencies. Thus, the difference between R_5_–R_20_ is considered a marker of small airway obstruction.^42^ Indeed, both R_5_ and X_5_ have been shown to be significantly increased in asthmatic patients, compared to healthy volunteers, while R_5_ shows a significant change after bronchodilation and is correlated with FEV_1_.^45^ Anderson et al^46^ have found that R_5_–R_20_ was increased in asthmatic patients across all stages (as defined by treatment steps), with the higher values observed in more severe stages. Recently in asthmatic patients, Abdo et al^47^ have shown that the presence of small airway disease, as indicated by an increased R_5_–R_20_, results in poor symptom control that negatively affects daily physical activity. Like resistance, reactance is also increased at lower frequencies (becomes more negative), and X_5_ is also used as a marker of small airways disease. Additionally, the area under the curve of reactance (A_x_) from the lowest frequency measured to the frequency where reactance is equal to zero (resonant frequency, *Fres*), has been used as a marker of small airway disease.^48^ In stable asthmatic patients, peripheral airway markers from oscillometry, i.e., R_5_–R_20_ and X_5_ correlated with quality of life and asthma control, as assessed by questionnaires, and retained their significant independent interaction, even when analyzed together with FEV_1_ and use of controller therapy in multiple regression analysis.^49^ The difference between inspiratory and expiratory reactance at 5 Hz (within-breath analysis) has also been used to differentiate asthma from COPD.^50^

Oscillometry has also been used to assess bronchodilator response,^51^^,^^52^ a hallmark of asthma pathophysiology and a marker of poor asthma control,^53^ and airway hyperresponsiveness during a bronchial challenge test, with cut-off values to define a positive response.^9^ Interestingly, baseline values of R_5_–R_20_ predict a higher response to metacholine challenge in asthmatic patients, when expressed as percentage fall of FVC (ΔFVC%).^54^ In subjects that developed symptoms during a metacholine challenge test, the onset of symptoms was associated with a significant increase in R_5_, while FEV_1_ change was minimal, indicating the role of peripheral airways contributing to patients’ symptoms.^55^ In asthmatic patients, the forced oscillation technique has been shown to be more sensitive than spirometry in detecting a positive bronchodilator response, and the change in reactance (higher ΔA_x_) post-bronchodilation was also associated with a poor asthma control test (ACT) score.^56^

The clinical applicability of oscillometry is also enhanced by the relatively low variability over time seen in a study that recruited moderate-to-severe asthma patients.^57^ Although changes in R_5_–R_20_ are caused by small airway disease,^58^ and the presence of peripheral inhomogeneities from small airway constriction,^9^^,^^25^ the frequency dependence of resistance can also arise from heterogeneities in more proximal airways, heterogenous time-constants due to airway obstruction and/or emphysema, tissue viscoelasticity, and upper airway shunt flow.^8^^,^^9^ A current limitation of oscillometry is that the use of different devices may lead to differences in measurements, especially at higher impedances.^59^ Indeed, to harmonise oscillometry technique, the European Respiratory Society has recently published technical standards.^9^ Oscillometry has also been used in the assessment of lung function in children with persistent asthma.^60^

### Inert gas washout techniques

In the single breath nitrogen washout (SBNW) technique, patients take a breath in to total lung capacity (TLC), while inhaling 100% of oxygen and exhaling to RV. Nitrogen concentration in the exhaled air is monitored and plotted against exhaled volume. The slope of the third phase (S_III_) of the N_2_ to volume diagram, known also as alveolar plateau, is used as a marker of ventilatory inhomogeneities in the lung.^10^ Moreover, SBNW technique enables the measurement of closing volume (CV) and closing capacity (CC), which are also indirect measures of small airways disease. In patients with severe asthma, the frequency of their disease exacerbation was associated with increased closing volume, indicating the important role of small airway closure in asthma exacerbation pathogenesis.^61^ Patients with allergic asthma and a high frequency of disease exacerbation (≥2/year) have been shown to have a greater S_III_, compared to asthmatic patients with fewer exacerbations.^62^ Multiple breath nitrogen washout (MBNW) is a technique that estimates ventilation distribution inhomogeneities by calculating nitrogen gas clearance during tidal breathing of 100% of oxygen.^10^ For each breath, the N_2_ tracing is recorded, and the phase III slope is computed, to estimate heterogeneities. Two main sources of inhomogeneities in ventilation distribution are present; inhomogeneities created in the conducting airways, i.e., airways proximal to terminal bronchioles (S_cond_) and inhomogeneities created in the diffusion-convection front, i.e., the zone where flow is converted from convection to diffusion (S_acin_), which is the acinar zone. Due to the differential time kinetics of S_acin_ and S_cond,_ i.e., S_acin_ reaches a steady value after the first breaths and then remains stable, partitioning of inhomogeneities to conducting airways and the more peripheral acinar zone, is then enabled.^63^ Lung Clearance Index (LCI), which is the ratio of the cumulative volume exhaled (CEV) to wash out the nitrogen to functional residual capacity (FRC) (LCI = CEV/FRC) can also be used as a global marker of inhomogeneities.

MBNW is a sensitive technique, able to detect underlying inhomogeneities even in patients with stable asthma,^64^ while its simple methodology, requiring only tidal breathing, enables its application in children.^65^ Using MBNW in mild asthmatic patients, Verbanck et al^66^ found increased heterogeneity in both the conductive and acinar airways. Interestingly, while salbutamol inhalation reversed heterogeneity in the acinar zone, the small conductive airways exhibited partial reversibility. In patients with severe acute asthma, both S_acin_ and S_cond_ were increased, although only heterogeneity in the acinar zone was correlated with FEV_1_.^67^ MBNW has also been found to be useful in predicting the response to treatment changes, since S_cond_ has been shown to be correlated with the asthma control questionnaire (ACQ)-5 following ICS up-titration in uncontrolled asthmatic patients.^68^ Although it has utility in assessing ventilatory inhomogeneities caused by small airways disease, currently MBNW is mainly available for research purposes. Moreover, compared to oscillometry, MBNW has shown moderate repeatability over time in asthmatic patients, and this requires further investigation to enforce clinical applicability.^57^

### Imaging

In asthma, the presence of expiratory gas trapping and mosaic pattern can be used as a surrogate marker of small airway disease.^69^ Measuring lung density at FRC or RV and counting voxels below a threshold (e.g. –850 hounsfield units [HU]) have been used in asthmatic patients to quantify gas trapping.^70^ Although direct visualization of the small airways is beyond the resolution of modern CT detectors, the use of novel algorithms has enabled the detection of small airway disease in obstructive airway diseases^71^ and imaging is expected to play an advanced role in the diagnosis and assessment of both asthma and chronic obstructive pulmonary disease (COPD) in the future.^4^^,^^72^ Indeed, advanced analysis of paired inspiratory–expiratory CT scans has produced imaging markers of small airway dysfunction, e.g., parametric response map (PRM), which allows the detection of the spatial distribution of small airway disease in asthma.^31^ Functional respiratory imaging (FRI) is another modern imaging technology that utilizes inspiratory and expiratory CT images, and is able to simulate airflow by applying computational fluid dynamics. FRI provides useful information not only for small airway disease but also for drug deposition.^73^ Hyperpolarized magnetic resonance imaging (MRI) using inhaled helium-3 or xenon-129 gas can also provide information on ventilation distribution and morphometry of the distal airways and lung parenchyma.^4^ In asthmatic patients, hyperpolarized helium-3 (He^3^) MRI has been used to detect ventilation defects that were correlated with disease severity and spirometric indices.^74^ Moreover, in a recent study, hyperpolarized MRI was used to assess and partition bronchodilator response between proximal and peripheral lung in patients with poorly controlled, moderate to severe asthma. Interestingly, peripheral small airways showed greater reversibility.^75^ Of note, He^3^-MRI use remains limited in research and is not widely available.

### Endobronchial optical coherence tomography

The combination of bronchoscopy with endobronchial optical coherence tomography (EB-OCT) is a novel and promising technique for the evaluation of the lung periphery, including both the alveolar space^76^ and small airways,^77^^,^^78^ while avoiding lung tissue sampling. EB-OCT uses near infrared light beams to illuminate tissue and detects back-scattered light and can achieve near-histological resolutions (1-20 µm range) with a penetration depth of few millimeters.^79^^,^^80^ In patients with asthma, EB-OCT revealed the presence of airway remodelling in small airways even in mild asthma, while EB-OCT imaging parameters correlated with disease severity.^81^

### Nuclear medicine

Using inhaled radiotracers and radio-labelled drug particles, the detection of ventilation distribution and drug deposition in the lung is enabled, by planar scintigraphy, three-dimensional single-photon-emission computer tomography (SPECT) or positron emission tomography (PET).^4^^,^^82^^,^^83^ In asthmatic patients, inhalation of radiolabeled monodisperse albuterol aerosol of varying mass median aerodynamic diameters (MMAD), revealed that regional distribution to proximal airways produced a larger increase in FEV_1_, compared to deposition in more distal airways, probably as FEV_1_ (a large airway marker) was related to the presence of large airways pathology, although there was greater deposition observed in the distal lung regions.^84^ The disadvantages of nuclear medicine imaging techniques are the exposure to ionizing radiation and that currently small airways are difficult to isolate.

### Biomarkers—exhaled

The detection of nitric oxide in exhaled breath (F_E_NO) is a marker of eosinophilic airway inflammation and is used in the diagnosis and assessment of asthma.^7^^,^^85^ Partitioning NO in a bronchial and peripheral (alveolar) components can be performed without the need of further equipment, by measuring F_E_NO during exhalations with two different flows.^86^ Indeed, the alveolar F_E_NO is correlated with asthma control in mild untreated asthmatic patients.^87^ Verbanck et al^64^ studied stable asthmatic patients, and showed the alveolar fraction of F_E_NO correlated with inhomogeneities in the acinar zone (S_acin_). The physical properties and content of exhaled particles are also under investigation for their potential role as markers of disease underlying mechanisms and site of particles generation.^88^

### Biomarkers—systemic

In asthmatic non-smoking patients under ICS treatment, blood levels of Clara Cell secretory protein (CC-16) were negatively associated with gas trapping, following a metacholine challenge test, indicating that reduced CC-16 levels could represent small airway disease in asthmatic patients.^89^ However, whether systemic biomarkers could be of value to detect small airway disease needs further evaluation.

## Special issues in small airways disease and asthma management

### Small airway disease in asthma diagnosis

Despite the emergence of data regarding the usability of oscillometry to detect early asthmatic disease, oscillometry or other markers specific to peripheral airway obstruction are not yet included in official asthma diagnostic algorithms.^7^ Assessing small airway function may offer significant advantages to the diagnostic pathway of asthma, as an adjunct to spirometry, given the increased sensitivity of both oscillometry and nitrogen washout techniques. In asthmatic patients with preserved spirometry (FEV_1_>80% pred), A_x_ was significantly increased, compared to non-asthmatics and the combination of spirometry with oscillometry showed increased overall diagnostic ability, compared to spirometry alone.^90^ Of note, the increased sensitivity of tools to assess small airway disease can be a double-edged sword, since it has been previously shown that 16% to 45% of healthy controls demonstrate signs of small airway disease with oscillometry (increased R_5_–R_20_), depending on the cut-off values used.^47^ This underlies the need of studies to establish normal reference values based on large populations.^91^ Reactance measured by oscillometry (both X_5_ and A_x_) showed also greater sensitivity, compared to spirometry (FEV_1_/FVC), in asthmatic patients presenting with symptoms and poor asthma control (ACT score<20), without loss in specificity.^56^

The absence of a need for co-operation increases the clinical utility of oscillometry in certain groups of patients such as children and the elderly. Indeed, Loeb et al^92^ have shown that the percentage of acceptable and repeatable spirometry depended on age, rising above only 50% by age of 6 years and reached a plateau with approximately 85% success at age of 10 years. This is very important given that relying only on clinical presentation without lung function testing results in a significant underdiagnosis of asthma in children.^93^ Avoiding forced expiratory maneuvers is also of value for patients with certain contra-indications for spirometry.^94^ Indeed, in a case series of patients with inherited connective tissue disorders and cardiovascular abnormalities for whom spirometry was deemed contra-indicated, impulse oscillometry was used as an alternative for asthma diagnosis and treatment modifications, with the R_5_–R_20_ marker being the most useful.^95^

Another clinical entity that assessing small airway disease could be of value is the diagnostic uncertainty of asthma in patients already receiving inhaled controller therapy (corticosteroids with or without bronchodilators), where classic spirometric indices may be within normal values, since according to GINA, the confirmation of asthma diagnosis requires variable airflow limitation.^96^ Indeed, Aaron et al^97^ recently showed that in a group of patients with physician-diagnosed asthma, a current diagnosis of asthma could not be established in 33.1% of patients who were not using daily asthma medications or had medications weaned, implying either asthma was in remission or an initial misdiagnosis. As previously stated, MBNW was able to detect underlying inhomogeneities even in patients with stable asthma under controller therapy,^64^ thus it may aid in asthma diagnosis in patients under treatment, avoiding an asthma medication step-down, which may impose the patients in the risk of asthma worsening.

### Multimodality studies

Given the complex pathophysiological mechanisms leading to small airway disease, it is highly unlikely that a single functional parameter will be able to describe the full spectrum of small airway dysfunction in asthma patients. Indeed, in uncontrolled asthmatic patients who were assessed by both impulse osillometry (IOS) and MBNW, baseline values of X_5_ and R_5_ had the strongest correlation with ACQ score, whereas S_cond_ was the best predictor of asthma control improvement, following a step-up in therapy.^98^ Interestingly, in this study, baseline values of oscillometry and MBNW were not correlated, which means that in this specific study population, the two techniques represented different aspects of small airway disease. Moreover, the combination of spirometry with techniques assessing small airway function may offer synergistic information, compared to each technique alone. Recently, it was shown that amongst asthmatic patients, impaired FVC and increased A_x_ were associated with the worst disease control and higher risk of exacerbations, compared with asthmatic patients with lower FVC but preserved A_x_.^99^ The prognostic value of combining spirometry with oscillometry was also shown in a retrospective study including moderate-to-severe asthma patients, where the group of patients with both reduced FEF_25%–75%_ and increased R_5_–R_20_, was characterized by worsening asthma control and increased disease exacerbation frequency, although not with blood eosinophilia.^100^ In contrast, a cross-sectional study in patients with both mild-to-moderate and severe asthma patients, including oscillometry and MBNW, failed to demonstrate a correlation of small airway function parameters, e.g., S_acin_, R_5_–R_20_ and A_x_, with asthma clinical outcomes.^101^ The authors speculated that the differences in methodology and performing lung function tests only post-bronchodilation may have affected the assessment of small airway function, resulting in opposite findings, compared to previously published studies.

Hitherto, the largest effort to provide a comprehensive assessment of small airways in asthmatic patients is the multicentre ATLANTIS study that evaluated lung physiology in asthmatic patients with a variety of methods and detected small airway disease by changes in the percentage decrease in FVC from baseline during a metacholine challenge test, forced expiratory flow (FEF_25%–75%_ and FEF_50%_ [spirometry]), RV/TLC and FRC (body plethysmography), R_5_–R_20_, A_X_, X_5_ (impulse oscillometry), conducting airway ventilation heterogeneity (S_cond_) and acinar airway ventilation heterogeneity (S_acin_) (MBNW).^102^ The initial cross-sectional analysis of the study revealed that small airway disease was present across all asthma severity stages, though its prevalence increased with more severe disease. Small airway disease was associated with less asthma control, older age, duration of asthma and frequency of exacerbation. Moreover, the group of patients with the higher small airway disease score presented also higher eosinophilic inflammation, as assessed by blood eosinophil levels. This agrees with findings in patients with persistent asthma, where small airway resistance, as assessed by IOS, was higher in patients with greater type-2 inflammation.^103^ The presence of small airway disease across all asthma severity stages is also confirmed in a recent systematic review of the literature that revealed a varying prevalence of small airway disease in asthma (ranging from 20% to 74%, with the vast majority around 50–60%), depending on the study population, the technique used and the set cut-off for normal values.^104^ Interestingly, in ATLANTIS, the prevalence of small airway disease was lowest when only S_acin_ was considered and a combination of oscillometry and spirometry provided a good discrimination between the different small airway disease groups.^48^ This finding supports the theory that small airway assessment should not be regarded as an alternative to classic lung function testing, e.g., spirometry and body plethysmography, but as an additive tool.

The association of small airway disease with asthma control and exacerbation was confirmed in a subsequent longitudinal analysis of ATLANTIS study.^105^ This study also showed that impulse oscillometry is an independent predictor of exacerbation frequency, even when other known predictors, i.e., previous exacerbations, GINA stage, FEV_1_% pred and blood eosinophils, are included in a multivariate analysis. Surprisingly, analysis of CT imaging failed to correlate with asthma control or exacerbations. An additional *post hoc* analysis of data from ATLANTIS study showed that physiological features of small airway disease, including R_5_–R_20_, S_cond_ and S_acin_, correlate with the presence of persistent airflow obstruction in asthma, a phenotype that was found not only in severe disease but also in mild asthma stages and correlated with asthma exacerbations frequency.^26^

### The role of small airways assessment in asthma therapy

Assessment of small airway function can play a dual role in asthma therapy, i.e., monitoring therapeutic efficacy on one hand and guiding the choice of aerosol therapy on the other hand. Indeed, given the association of small airways with important clinical outcomes, e.g., asthma control, quality of life and exacerbation frequency, markers of small airway dysfunction could serve as biomarkers and surrogate endpoints in asthma clinical trials. For example, in patients with asthma that were currently smoking, adding dual bronchodilation (olodaterol/ tiotropium) to ICS, resulted in significant improvement in small airway resistance, as assessed by oscillometry, compared to single bronchodilation (olodaterol).^106^ Moreover, in patients with poorly controlled asthma, changes inS_cond_ and S_acin_ were the only independent variables of patients’ response to inhaled corticosteroids.^107^ In addition to inhalational therapy, physiology of small airways has also been used to monitor the efficacy of systemic asthma therapy. Kraft et al^108^ reported that in patients with mild asthma, oral montelukast resulted in significant reduction of RV, denoting the improvement in peripheral airway function. Moreover, in patients with severe eosinophilic asthma, add-on therapy with the anti-IL-5 antibody, mepolizumab, resulted in a significant improvement of small airways function, as assessed by either the forced oscillation technique (R_5_, X_5_)^109^ or the multiple breath nitrogen washout (LCI, S_acin_).^110^ The above findings were confirmed in a real-world study, where add-on biology therapy with either anti-IgE (omalizumab) or anti-IL-5 (mepolizumab, benralizumab) in severe asthma patients, resulted in an overall significant increase in FEF_25%–75%_, and a reduction of R_5_–R_20_ in those patients that presented initially with increased peripheral airway resistance.^111^ There is a need to undertake real-world evidence-based studies in the area of small airways.^112^

Given the key role of lung periphery in asthma, therapies targeting small airways provide a promising treatment option.^113^ The most important factors of inhaled drug formulations that determine the deposition and regional distribution of inhaled particles are the particle size (mass median aerodynamic diameter [MMAD]) and the disperse of particles within an aerosol.^114^ Indeed, using gamma scintigraphy, it has been shown that for both β2-agonists (salbutamol) and inhaled corticosteroids (ciclesonide), small particles (<2 µm MMAD^115^) achieve a greater deposition in the lung periphery and small airways and a lower deposition in oropharynx.^84^^,^^116^ In patients with stable atopic asthma under ICS with a dry powder inhaler (DPI) but with peripheral inhomogeneities (increased S_acin_), switching to ultra-fine ICS therapy caused a reduction in ventilation inhomogeneity at the acinar level.^117^ In contrast, in patients with well-controlled asthma, but acinar ventilation inhomogeneities, as assessed by increased S_acin_, switch of non-fine particle ICS therapy, to fine particle ICS failed to reverse inhomogeneities.^118^ Moreover, therapy with inhaled extra-fine ciclesonide was associated with improvement in small airway function, measured by oscillometry, and peripheral airway inflammation, as assessed by late-phase sputum eosinophilia, compared to a non-extra fine ICS treatments.^119^ In terms of clinical outcomes, in literature, it has been shown that small-size particle inhalational therapy achieves at least equal, and even greater efficacy in some studies, compared to larger particles, allowing also a reduction in total inhaled corticosteroid dose.^115^^,^^120^^,^^121^ For example, Postma et al^122^ reported that in asthmatic patients, initiating treatment with extra-fine ciclesonide resulted in better clinical outcomes (e.g., asthma control, exacerbation frequency), compared to non-extra fine ICS. Correspondingly, evidence from a real-world observational study suggested that initiating or stepping up asthma therapy with extra-fine beclomethasone achieved better asthma control, compared to large-particle beclomethasone, in spite using lower doses.^123^ It should be clarified that studies investigating drug deposition in the lung have shown that small particle aerosols show increased peripheral deposition, but also achieve adequate deposition in larger airways, which is important given that asthma is a disease of the “whole” airway tree,^124^ and drug targets (e.g., β2-receptors and muscarinic receptors) are localised in both large and small airways.^125^ Indeed, the recent STORM study, evaluating the deposition of extrafine triple therapy with beclomethasone/formoterol and glycopyrronium via a pressurized metered-dose inhalers (pMDI) using gamma-scintigraphy, showed that the lung dose was distributed almost equally between the peripheral and central part of the lung, a pattern that was the same for both healthy volunteers and asthmatic patients.^126^

## Conclusion

Cumulative evidence from both experimental and clinical studies has proved the significant role of small airways in asthma pathogenesis and pathophysiology. Although in literature, it is suggested that specific characteristics of asthmatic patients are associated with increased small airway dysfunction (e.g., nocturnal asthma, T_2_-high inflammation, fixed airflow limitation),^39^ it should be emphasized that small airway disease is a hallmark characteristic of asthma in general, with significant prevalence across all asthma stages and especially severe disease. As discussed in this review, small airway disease affects important clinical outcomes of asthma, such as disease control, quality of life and exacerbation frequency. Standardization of techniques specific to small airway assessment, such as oscillometry, and establishment of robust reference equations, will enable their incorporation into diagnostic pathways and can provide valuable information for asthma diagnosis and monitoring. Detection of small airway disease could also be part of a precision medicine approach in asthma treatment and guide aerosol therapy.^127^ Finally, inclusion of markers of small airways disease as endpoints in large clinical trials is needed to establish their role in therapeutic algorithms.

## Conflicts of interest

The authors declare the following financial interests/personal relationships which may be considered as potential competing interests:

Omar Usmani reports a relationship with Astra Zeneca that includes: consulting or advisory, funding grants, speaking and lecture fees, and travel reimbursement. Omar Usmani reports a relationship with Boehringer Ingelheim that includes: consulting or advisory, funding grants, speaking and lecture fees, and travel reimbursement. Omar Usmani reports a relationship with Chiesi Pharmaceuticals Inc that includes: consulting or advisory, funding grants, speaking and lecture fees, and travel reimbursement. Omar Usmani reports a relationship with Covis Pharma that includes: consulting or advisory, speaking and lecture fees, and travel reimbursement.

Dimitrios Toumpanakis reports a relationship with Chiesi Pharmaceuticals Inc that includes: speaking and lecture fees. Dimitrios Toumpanakis reports a relationship with Menarini that includes: speaking and lecture fees. Dimitrios Toumpanakis reports a relationship with Elpen Pharmaceuticals that includes: speaking and lecture fees.
